# Field-based evidence of fast and global increase of *Plasmodium falciparum *drug-resistance by DNA-microarrays and PCR/RFLP in Niger

**DOI:** 10.1186/1475-2875-8-32

**Published:** 2009-02-23

**Authors:** Maman Laminou Ibrahim, Nicolas Steenkeste, Nimol Khim, Hadiza Hassane Adam, Lassana Konaté, Jean-Yves Coppée, Fredéric Ariey, Jean-Bernard Duchemin

**Affiliations:** 1 Centre de Recherche Médicale et Sanitaire (CERMES), BP 10887 Niamey, Niger; 2 Unité d'épidémiologie moléculaire, Institut Pasteur, BP 983, Phnom Penh, Cambodia; 3 Faculté des Sciences et Techniques, UCAD, BP 5005, Dakar, Sénégal; 4 Institut Pasteur, Plate-forme 2, Puces à ADN, 25-28 rue du Docteur Roux, 75015 Paris, France

## Abstract

**Background:**

Over the last years, significant progress has been made in the comprehension of the molecular mechanism of malaria resistance to drugs. Together with *in vivo *tests, the molecular monitoring is now part of the survey strategy of the *Plasmodium *sensitivity. Currently, DNA-microarray analysis allows the simultaneous study of many single nucleotide polymorphisms (SNP) of *Plasmodium *isolates. In December 2005, the International Federation of the Red Cross distributed two million three hundred thousand long-lasting insecticide nets to pregnant women and mothers of under five years children in the whole Niger. Then, Niger adopted artemisinin-based combination therapy as first-line treatment.

**Methods:**

Thirty four SNPs of *pfcrt, pfdhfr, pfdhps, pfmdr *and *pfATPase *were analysed by DNA-microarray and PCR/RFLP in two villages – Zindarou and Banizoumbou – with different durations of malaria transmission. The main objective of the study was to measure the dynamics *of Plasmodium falciparum *resistant strains and associated factors.

**Results:**

This study shows a global and clear increase of the drug-resistance associated molecular markers frequencies during a relatively short-time period of four years. Markers associated with resistance to chloroquine and sulphonamids were more frequently found in the short transmission zone than in the long transmission one. The *pfcrt76T *mutation is significantly more present at Banizoumbou than Zindarou (38.3% vs 25.2%, p = 0.013).

This work allowed the screening of several field strains for five SNPs of *PfATPase6 *gene. The *pfATPase6S769N*, candidate mutation of resistance to artemisinin was not found. However the *pfATPsaeA623E *mutation was found in 4.7% of samples.

**Conclusion:**

A significant increase of several SNPs frequencies was highlighted over a four-year period. The polymorphism of five *PfATPase6 *gene SNPs was described. The global, large and fast increase of the molecular resistance is discussed in the context of current changes of health policy and malaria control in Niger.

## Background

Malaria drug resistance is a major world wide public health issue. If funding efficacious drugs is presently possible for many African countries, the future remains questionable. For many transmissible diseases, the efficacy of chemical treatment is a matter of time and resistant strains can emerge [1]. If regularly monitoring the pattern of drug resistance is of prime importance, questioning the processes involved in the emergence of resistance and selection helps to define the treatment policy. *Plasmodium falciparum *has been shown to use several mechanisms for drug resistance. These last decades, the knowledge about molecular mechanisms of malaria drug resistance has significantly increased. Molecular surveys are now included in the *P. falciparum *sensitivity surveillance strategy promoted by the WHO, in association with *in vivo *tests. The resistance to chloroquine is linked to the *pfcrtK76T *mutation, a gene located on chromosome 7 [2], and clinical assays have confirmed this association [3,4].

The *dhfrS108N mutation*, carried by chromosome 4, is the key mutation site for resistance to pryrimethamine. However, the mutations of the SNPs 51 and 59 modulate it. The triple mutation *dhfrS108AN, dhfrC59R, dhfrN51I *have been shown to be selected in failures of the sulphadoxine-pyrimethamine association (SP) [5,6]. The *pfdhps *gene on chromosome 8 is coding for the dihydropteroate synthetase (*pfdhps*) and mutations of this gene are linked to sulphamid resistance. The *dhpsS436A, dhpsA437G, dhpsK540E and dhpsA581G *mutations have been described [4,7]. The *dhpsA437G*, *dhpsK540E *double mutation or the *pfdhpsS436A*, *dhpsA437G*, *dhpsK540E *triple mutation are found linked with the higher resistance levels [5,7].

The multi-drug resistance gene of *P. falciparum*, localized on chromosome 5, is coding for the P-glycoprotein. The *pfmdrA86Y *mutation is suspected to be linked with the cross-resistance to amodiaquine and chloroquine [8,9]. The copy number of *pfmdr *gene is suspected to be associated to mefloquine resistance [10]. The SERCA-*pfATPase6 *gene is a putative candidate to the artemisinin derivatives resistance. The *pfATPase6S769N mutation *has been described associated with artemether increased CI_50 _values in French Guyana [11]. However, this mechanism does seem to be unique, since *Plasmodium *may develop artemisinin resistance without that mutation [12].

Usually, the PCR-RFLP is used to study these mutations. Even if robust and affordable for African malaria research centres, this method does not allow a high throughput screening of many mutations sites. The development of micro-array techniques offers the ability of such a screening of many isolates [13], including field-collected ones [14]. So the monitoring of *P. falciparum *sensitivity to treatment survey can take benefit of this technique.

In Niger, malaria remains the main cause of morbidity and mortality with about 850,000 declared cases a year, an estimated incidence of 419/1,000 inhabitants and an estimated global fatality rate of 0.56%[15], reaching 20% in hospitalized patients [16].

However, Niger has entered in a very active phase of malaria control. A mass distribution of long-lasting insecticide-impregnated bed nets to mothers of children under five has been implemented in December 2005. Pregnant women benefit from intermittent preventive treatments with sulphadoxine-pyrimethamine (SP). Finally, Niger has adopted the use of artemisinin combination therapy (ACT) as first-line treatment of uncomplicated malaria cases. In this context, it is of prime importance to follow the sensitivity of *P. falciparum *strains to treatment, both old ones and new ones. Beside *in vivo *studies led by the National Malaria Control Programme (PNLP) and WHO-Niger [17], the CERMES has set up a molecular survey programme [18]. Two molecular markers have been studied by par PCR-RFLP: *pfcrt76 *and *pfdhfr108*. In the Niger river valley, the prevalence of the *pfcrt76T and dhfr108N *mutations was respectively 50.8% and 57% in 2005 [19]. In collaboration with the Institute Pasteur of Cambodia, some of the samples were tested with DNA-microarray dedicated to the detection of Single Nucleotide Polymorphisms (SNPs) associated with *P. falciparum *resistance [20,14].

To explore the conditions of emergence and spread, the temporal evolution of molecular markers of *P. falciparum *drug resistance was studied in two Sahel villages between 2003 and 2006. Usual old drugs-chloroquine and pyrimethamine – were tracked by *PfcrtK76T *and *pfdhfrS108N *mutations by the microarray and PCR-RFLP methods, while other potential molecular markers from five genes, *pfcrt, pfdhfr, pfdhps, pfmdr and pfATPase*, were studied only by the microarray technique.

## Materials and methods

### Study sites

**Figure 1:**
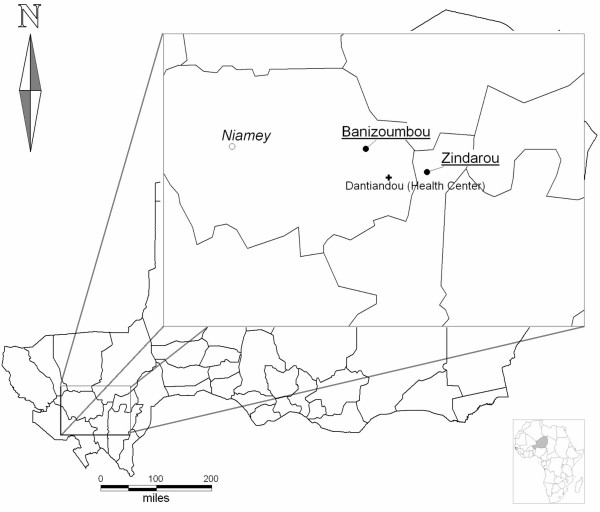
**Study sites**. Map of Niger with the larger insert for the study zone showing the relative situation of villages to the Capital, Niamey. Scale bars for the Niger map.

Zindarou (13°26.09 N/2°55.23 E) and Banizoumbou (13°32.03 N/2°39.66 E) are two villages of comparable size (respectively 500 and 1,000 inhabitants), 70 km far from Niamey and 20 km distant from each other (Figure 1). If the rainfall is considered to be equivalent, the land use and topographies of the two villages determine different length and intensities of malaria transmission. The malaria transmission is short (three months) and strictly linked to rainy season in Banizoumbou, while it goes beyond five to six months in Zindarou with presence of *Anopheles funestus *[21], in addition to *Anopheles gambiae sl*.

### Study design

Samples from asymptomatic *P. falciparum *carriers were tested from two villages (46 samples each). Patients were stratified by age (under 15 and over 15 years old) and by parasitaemia (46 patients presenting parasitaemia under 500 parasites and 46 over 500 parasites/μl). The two years of collection, 2003 and 2006, were equally distributed (46 patients sampled in 2003 and 46 in 2006). The collections were performed in October, at the end of the rainy seasons. These samples were tested by DNA micro-array technique, targeting the global temporal evolution between the two years 2003 and 2006 for five genes. These data were completed by testing of additional samples from the same two villages, respectively 250 and 245 samples in Zindarou and Banizoumbou, collected during the four years from 2003 to 2006. The aim of these extra materials was to see if the temporal trend had a continuous or a discontinuous pattern. These last samples were tested by PCR-RFLP for the only *pfcrtK76T *and the *pfdhfrS108N *mutations, which both presented the most evident temporal evolutions by DNA micro-array technique. The National Ethics Committee of Niger approved the study.

### Methods

Blood slides and finger pricks on filter cards were performed in the field. Parasite carriage was assessed by microscopy after Giemsa staining. The parasitaemia threshold was established to 40 parasites/μl. DNA was extracted in 96 well-plates by resin method with Instagen. Briefly, red blood cells were discarded from filter cards after lysis with HBS1x/10% saponin (Hepes 100 mM, NaCl 1.4 M, KCl 100 mM) buffer then washed three times. Parasites were heated at 56°C then 90°C with 200μl of Instagen. Finally DNA was purified by centrifugation at 4,000 rpm for 20 mn.

The PCR-RFLP methods targeting *pfcrt76 *and *pfdhfr108 *followed the procedures described by Djimdé *et al *[3] and Kublin *et al *[5].

The microarray method was used as described in Crameri *et al *[20]. This method was performed at the Institute Pasteur of Cambodia. A total of 34 SNPs from five different genes (*pfcrt, pfdhfr, pfdhps, pfmdr and pfATPase*) were analysed by 34 SNPs. Decision values were determined by an algorithm including absolute signal and relative (green/red) values of optical densities [22]. Control samples with known SNPs patterns were introduced in the assay.

### Statistical analysis

The results were transferred to a database and analyzed by EPI-Info™ (Centers for Disease Control and Prevention). The mixed (wild + mutation) isolates were encoded as mutation. The bivariate analysis was performed with Epi-Info. Once found at p 0.20 significance threshold, the independence of factors (age, sex, parasitaemia, village and year) was tested by a conditional backward stepwise logistical regression model with molecular markers as dependant variables with the SPSS software (SPSS Inc., Chcago).

## Results

### Samples characteristics

495 samples were tested by PCR/RFLP, among which 250 from Zindarou and 245 from Banizoumbou villages. 92 samples were tested by DNA-chips method with 46 samples from each village. The mean age was 11.26 yrs old (N = 587). The sex ratio (F/M) was 1.14 (N = 587). The mean geometrical mean of parasitaemia was 1.364 parasites by μl (N = 587).

### Polymorphisms and prevalence of mutations

The data are presented gene by gene. The analysis was performed on data following quality criteria, i.e. above optical density thresholds.

#### *pfcrt *gene

From the 12 SNPs used for the study of the *pfcrt *gene, two did not give data for analysis (*pfcrtQ271E *and *pfcrtN326S*). Among the remaining 10 *pfcrt *SNPs, five (*pfcrt72*, *pfcrt75*, *pfcrt152*, *pfcrt163*, *pfcrt356*) did not show any polymorphism and thefive others were polymorphic at the 74, 76, 97, 220 and 371 codons (Table 1). All methods mixed, DNA micro-array and PCR/RFLP, the mutation *pfcrtK76T *prevalence was 32.4%.

**Table 1:** Relative frequencies of polymorphic *Pfrct *gene SNPs according to villages, ages and years

	**Number of samples**	**Overall**	**Banizoumbou vs Zindarou**	**Adults vs under 15**	***2003 vs 2006***
	
*pfcrtMet74IIe*	*87*	10.3%	11.1% vs 9.5% (p = 1.0)	22.2% vs 7.1% (p = 0.084)	0.0% vs 21.4% (**p = 0.001**)*
*pfcrtLys76Thr*	*318*	32.4%	38.3%vs 25.2% (**p = 0.013**)*	37.8% vs 31.5% (p = 0.405)	20.0% vs 44.4% (**p = 0.013**) *
*pfcrtHis97gln*	*87*	11.5%	15.6% vs 7.1% (p = 0.317)	16.7% vs 10.1% (p = 0.425)	2.2% vs 21.4% (**p = 0.006**)
*pfcrtAla220Ser*	*77*	10.4%	10.0% vs 10.8% (p = 1.0)	25.0% vs 6.5% (p = 0.053)	0.0% vs 19.5% (**p = 0.006**)*

in bold p < 0.05

* link kept in logistical regression model)

Global trend for samples from 2003 to 2006 years for *pfcrt76 *SNP gene

#### *pfdhfr *gene

Five SNPs targeted the *pfdhfr *gene. Among them, one (*pfdhfrI164L*) did not give sufficient quality for analysis and another (pf*dhfr16*) was monomorphic. The three remaining sites were polymorphic: *pfdhfrS108N*, *pfdhfrC59R *and *pfdhfrN51I *with respective prevalences of 53.8%, 76.7% and 21.2%. The 51–59 double mutation was present in 23.2% and the 51–59–108 triple mutation in 11.8% of samples (Table 2).

**Table 2:** Relative frequencies of polymorphic *Pfdhfr *gene SNPs according to villages, ages and years

	**Number of samples**	**Overall**	**Banizoumbou vs Zindarou**	**Adults vs under 15**	***2003 vs 2006***
	
*pfdhfrAsn51IIe*	85	21.2%	22.7% vs 19.5% (p = 0.717)	23.5% vs 20.6% (p = 0.75)	9.3% vs 33,3% (**p = 0.008**)
*pfdhfr Cys59Arg*	56	76.7%	80.0% vs 73.1% (p = 0.541)	77.8% vs 76.6% (p = 0.939)	73.9% vs 78,8% (p = 0.671)
*pfdhfrSer108Ans*	286	53.8%	58.4% vs 46.9% (p = 0.057)	40.0% vs 56.1% (p = 0.058)*	21.7% vs 45.7% (**p = 0.015**)*
*51,59*	56	23.2%	20.0% vs 26.9% (p = 0.541)	44.4% vs 19.1% (p = 0.189)	4.3% vs 36.4% (**p = 0.008**)*
*51, 108*	81	11.1%	12.2% vs 10.0% (p = 1.0)	14.3% vs 10.4% (p = 0.65)	4.7% vs 18.4% (p = 0.076)
59,108	56	30.3%	40.0% vs 19.2% (p = 0.092)	33.3% vs 29.8% (p = 1.0)	7.4% vs 39.4% (p = 0.139)
51, 59,108	56	11.8%	13.3% vs 11.5% (p = 1.0)	22.2% vs 18.2% (p = 0.312)	0% vs 21.2% (**p = 0.034**)

in bold p < 0.05

* link kept in logistical regression model)

Samples from 2003 to 2006 years for *pfdhfr108 *SNP gene

#### *pfdhps *gene

Four SNPS presented polymorphisms in the *pfdhps *gene at the 436, 437, 581 and 640 positions. The prevalence of *pfdhpsS436F*, *pfdhpsA437G *double mutation was 61.2%. The *pfdhpsSer436Phe*, *pfdhpsA437G *and *pfdhpsA640T *triple mutation was present in 58.3% of cases and the *pfdhpsS436F*, *pfdhpsA437G*, *pfdhpsA581G*, *pfdhpsA640Y *quadruple mutation in 10.6% (Table 3). The *pfdhpsK540G *mutation was not found. No meaningful result was obtained for the *pfdhpsI613F *codon. The codons *dhps540 *and *dhps645 *did not show any polymorphism.

**Table 3:** Relative frequencies of polymorphic Pfdhps gene SNPs according to villages, ages and years

	**Number of samples**	**Overall**	**Banizoumbou vs Zindarou**	**Adults vs under 15**	***2003 vs 2006***
	
*pfdhpsSer436Phe*	84	71.4%	73.8% vs 69.0% (p = 0.629)	52.9% vs 76.1% (p = 0.059)	76.2% vs 66.7% (p = 0.334)
*pfdhpsAla437Gly*	49	73.4%	71.0% vs 77.8% (p = 0.743)	50.0% vs 79.5% (p = 0.06)	77.3% vs 70.4% (p = 0.586)
*pfdhpsAla581Gly*	79	10.1%	19.0% vs 0.0% (**p = 0.006**)*	12.5% vs 9.5% (p = 0.661)	0.0% vs 20.5% (**p = 0.002**)*
*pfdhpsAla640Thr*	90	97.8%	95.6% vs 100.0% (p = 0.494)	100.0% vs 97.1% (p = 1.0)	100.0% vs 95.5% (p = 0.236)
*436, 437*	49	61.2%	61.3% vs 61.1% (p = 0.99)	30.0% vs 69.2% (**p = 0.033**)	68.2% vs 55.6% (p = 0.367)
*436, 437, 581*	48	10.4%	16.1% vs 0.0% (p = 0.146)	10.0% vs 10.5% (p = 1.0)	0.0% vs 19.2% (p = 0.054)
*436, 437, 640*	48	58.3%	56.7% vs 61.1% (p = 0.762)	30.0% vs 65.8% (p = 0.07)*	50.0% vs 68.2% (p = 0.203)
*436, 437,581, 640*	47	10.6%	16.7% vs 0.0% (p = 0.143)	10.0% vs 10.8% (p = 1.0)	0.0% vs 20.0% (p = 0.052)

in bold p < 0.05

* link kept in logistical regression model

#### *pfmdr *gene

The two positions 86 (17.4%) and 1034 (4.6%) of the *pfmdr *gene were polymorphic, but no double mutants was found (Table 4). The codons *pfmdrY184F *and *pfmdrN1246Y *did not give sufficient quality results to allow the analysis. The *pfmdr1042 *position was found monomorphic.

**Table 4:** Relative frequencies of polymorphic *Pfmdr and pfATPase *genes SNPs according to villages, ages and years

	**Number of samples**	**Overall**	**Banizoumbou vs Zindarou**	**Adults vs under 15**	**2003 vs 2006**
	
*pfmdrAsn86Ser*	86	17.4%	13.6% vs 21.4% (p = 0.341)	5.9% vs 20.3% (p = 0.284)	22.7% vs 11.9% (p = 0.186)
*pfmdrSer1034Cys*	86	4.6%	6.7% vs 2.4% (p = 0.618)	12.5% vs 2.9% (p = 0.156)	0.0% vs 9.8% (**p = 0.048**)*
*pfATPaseAla623Glu*	86	4.7%	6.8% vs 2.4% (p = 0.616)	5.6% vs 4.4% (p = 1.0)	0.0% vs 9.5% (p = 0.053)

in bold p < 0.05

* link kept in logistical regression model

#### *pfATPase6 *gene

Only the 623 codon of the *pfATPase6 *gene was polymorphic and the mutation was found in 4.7% (4/86) of the cases (Table 4). The *pfATPaseS769N *mutation was not found as were not the others targeted mutations at the *538, 574, and 683 *codons.

### Mutations associations

Only three strains presented both a mutation at *pfmdr *and *pfcrt *genes (two with *pfmdr86 *and *pfcrt76 *and one with *pfmdr1034 *and *pfcrt76*). All these three isolates came from 2006 samples.

Considering the *pfdhfr *and *pfdhps *genes, targeting the SP efficacy, one strain presented both the triple 51, 59 and 108 *pfdhfr *mutation and the quadruple 436–437–581 and 640 *pfdhps *mutation. Among the triple *pfdhfr *mutants, seven isolates presented at least one mutation at the *pfdhps *gene; all of them were collected in 2006.

### Variations according to time

**Figure 2:**
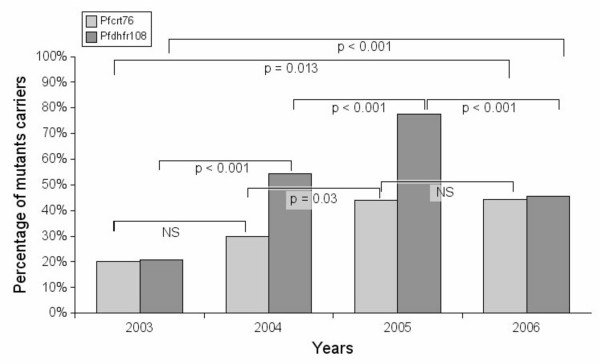
**Yearly evolution of pfcrt 76 (light grey) and pfdhfr 108 (dark grey) molecular markers**. P coefficients correspond to chi-square two tailed tests.

All the five polymorphic codons of the *pfcrt *gene presented a significantly increased prevalence between 2003 and 2006. For the *pfcrt76T *mutation, the year-to-year evolution showed the same trend (*Kruskall-Wallis df 3, p = 0.013*) with the 2004/2005 transition statistically significant (p < 0.05): 20% (2003), 27.8% (2004), 43.9% (2005) and 41.8% (2006) (Figure 2).

For the *pfdhfr *gene, the mutations at positions 51 (p = 0.008) and 108 (p = 0.015) have also significantly an increased prevalence between the two years 2003 and 2006. The prevalence of the *pfdhfrS108N *mutation increased significantly from year to year (*Kruskall-Wallis df 3, p < 0.001*) except between 2005 and 2006 where the prevalence decreased significantly (p < 0.001). Between 2003 and 2006, the prevalence of the 51/59 double mutation increased significantly from 4.3% to 36.4% (p = 0.008) as the 51/59/108 triple mutation from 0% to 21.2% (p = 0.034) (Table 2).

For the two *pfdhps *and *pfmdr *genes presenting respectively four and two polymorphic codons, only the prevalences of *pfdhpsA581G *(0% vs. 20.5%, p = 0.002) and *pfmdrS1034C *(0% vs 9.8%, p = 0.048) mutations have significantly increased between 2003 and 2006. All samples with the *pfATPaseAla623Glu *mutation were found during 2006 (p = 0.053). Globally, among the all 15 polymorphic codons, the prevalence of nine were significantly increasing between the 2003 and 2006 years, when no mutation prevalence was significantly decreasing.

### Variation according to the villages (malaria transmission length)

Globally, the *pfcrt76T *mutation was significantly more present at Banizoumbou than Zindarou (38.3% vs. 25.2%, p = 0.013) (Table 1). The other polymorphic mutation positions (74, 97, 220 and 371) did not differ between locations. For the *pfdhfr gene*, the *108 *(58.4% vs. 46.9%), mutation and the 108/59 (40.0% vs. 19.2%) double mutation had the same trend as *pfcrt *– i.e. higher in Banizoumbou – but none significantly. For the *pfdhps gene*, only the *pfdhpsA581G *mutation was significantly different in the two villages, being present only at Banizoumbou (19% vs. 0%, p = 0.006). The frequencies of mutations of the two genes *pfmdr *and *pfATPase *were not different in the two villages.

### Variation according to host factor (age, sex, parasitaemia)

The only significant link found was the under 15 years age more often associated with the double *pfdhps *436–437 mutation (Table 3). No link was found between the studied mutations, parasitaemia or sex.

## Discussion

The data show a global and significant increase of the drug resistance associated molecular markers frequencies. This increase concerns several (9 out of 15) polymorphic molecular markers linked to different metabolic pathways. The study covered a relatively short-time period of four years, showing a mean doubling of frequencies of mutations highly linked to resistance, such as *pfcrt76 *and *pfhdfr108*. The dynamics highlighted here appear to be fast, global and intense.

These data add arguments to the debate about the links between transmission and resistance [23,24]. On the one hand, Talisuna *et al *in Uganda [24] have shown higher levels of resistance to chloroquine and SP in zones of higher transmission intensity. On the other hand, history shows that chloroquine resistance emerged first in low transmission zones and that antifolate resistance has increased more rapidly in low transmission areas [25]. Theoretical models support the view that the spread of resistance in low transmission area is favoured either by the high selfing-rate [23] or by intra-host competition [24]. Others models [26] have shown that the transmission rate may not be the main force driving the resistance spread, which may rather be led by drug pressure or immunity.

However, field data are scarce. Here, the mutations tended to be more frequent in the village with the shorter malaria season: Banizoumbou. This was verified for the *pfcrtK76T *mutation. It has to be noted that the two villages share very comparable social patterns (distance from main road, health facilities, ethnicity, millet-based economy), but differ in their water bodies – ie *Anopheles *breeding sites – duration times. This corroborates the hypothesis that the resistance could spread more easily in low-transmission zones. The field data presented here correspond to one of the most restricted spatial scale ever described for this effect: these two villages are only 30 km distant. History has shown at the worldwide-scale that resistance emerged from low transmission area (Amazonia, South East Asia), and this may be verified at a very local scale. The same tendency for *pfdhfr *and others markers seems to give a global value to the processes involved in the spread of resistance.

The frequencies of mutation tended to be higher among the younger parasite carriers. However, this relation was significant only for the *dhps436*/*437 *double mutation. As the immunity or the drug pressure may be involved, the study was not designed to answer these issues. The global prevalence of *pfcrtK76T*, 32.4%, was lower than in many published works performed in Africa [27], but not so far from regional data [3,28]. In comparison with local data this prevalence was different from the one observed in hospitalized urban population recorded in 2003: Ibrahim *et al *found respective prevalence of *pfcrtK76T *and *pfdhfrS108N *mutations of 45.4% and 61.9% [18]. Later in 2005 [19] among patients attending rural health centres, the prevalence was closer to the 2005 values: 50.8% for *pfcrt76 *and 57.7% for *dhfr108*. However, the present work was targeting asymptomatic carriers in a rural zone, not patients attending to clinics or hospitals, and the drug pressure appears to be different in those different populations.

Recent works have studied the *ATPase6 *gene and specially the *pfATPase6S769N *mutation as a candidate marker for artemisinin resistance [11]. For first time, several field strains could be screened for five SNPs of *PfATPase6 *gene. The *pfATPase6S769N *mutation was not found. However, the *pfATPaseA623E *mutation was found in 4.7% of samples. The more precise sequence data for this gene are presented elsewhere [29].

The respective factors driving the emergence and spread of malaria drug resistance are not well known. Drug pressure, clonality of infections, pharmacokinetics, human migrations, immunity, fitness of parasites and transmission levels have all been suspected [24-27,30,31]. One interesting point of this work is the role of the intervention to block transmission. Two millions and three hundred thousands long-lasting insecticide-impregnated bed nets were distributed in December 2005. The bed nets reduce the lifespan of the vectors and the contact between human host and vector. This decreases the malaria transmission. Alifrangis *et al *have observed a significant decrease of molecular resistance to pryrimethamine after impregnated bed nets in Tanzania [32]. On the other hand, a controled trial of the impact of impregnated bed nets in Ivory Coast found no significant difference in *pfcrt, pfdhfr *and *pfdhps *mutations prevalences between treated villages and negative controls [33,34]. Diallo *et al *in Burkina Faso observed that impregnated curtains are not associated with an increase of molecular resistance or therapeutic failure in children under five years of age [35].

A decrease or stabilization of the molecular resistance has been shown between 2005 and 2006 for *pfcrtK76T *and *pfdhfrS108N *(Figure 2), when the trend is to a global increase during the 2003–2006 period. During this period, given the parasite carriage among children between two and five years of age, the malaria transmission in the two villages has decreased significantly (chi-square p < 0.0001), whether due or not to the impregnated bed nets distribution, from 56.5% (n = 255) in 2005 to 37.1% (n = 237) in 2006. What was observed in molecular markers then could be related either to a decrease of drug pressure or to a direct effect of transmission decrease on mutation carrying strains. A decrease of drug pressure cannot account for the *pfdhfrS108N *dynamics, when the SP is not often used in the study zone and rarely as the first-line treatment [18]. Beside this drug pressure hypothesis, the decrease of resistant strains may be related to:

• An absolute – i.e. in absence of drug pressure – weaker fitness of resistant strains in competition with wild strains during the transmission season: this is compatible with the observed higher frequency of resistant strains in low transmission area, where the competition between strains related to the chance of transmission events, is lower.

• A best fitness of mutation carrying strains, when drugs are delivered during the malaria season, with a relative lower fitness of wild strains, especially at the end of the transmission season when the drug pressure is maxima. This effect is cumulative during the dry season, when there is no transmission and thus no competition with wild strains. So the resistance could favour the longer parasite carriage during the dry season, but not the competition with wild strains as the new transmission season begins. This theoretical view is in accordance of the observed decrease after bed net distribution by personal protection and decrease of drug pressure.

• Whatever the processes involved, and taking in account the short duration time of the malaria transmission period in Sahel, the selective advantage kept all along the dry season by mutation carrying strains seems to be cumulative from year to year and tends to a global increase.

## Conclusion

This work highlights a global increase of frequencies of mutation molecular markers linked to malaria drug resistance during a relatively short period of four years. Given the wide range of molecular markers studied by the DNA microarray method, the observed increase of several markers corresponds to different metabolic pathways. This reinforces the hypothesis of a global process involving transmission and fitness of mutation, rather than simultaneous drug pressures. In the context of new reinforcement of malaria control in Niger, based both on introduction of artemisinin-based combination therapy and vector control, the present data may be considered as baseline data for future drug resistance monitoring.

## Competing interests

The authors declare that they have no competing interests.

## Authors' contributions

MLI carried out the molecular genetic studies and drafted the first manuscript version. HHA participated to fieldwork and DNA extraction. NK and NS participated in the DNA-microarray assays and algorithm. LK participated to draft improvement. JYC contributed to the DNA microarray design. FA and JBD conceived the study, participated in its design and coordination and elaborated the final version of manuscript. All authors read and approved the final manuscript.
